# Síndrome de wolff parkinson white en un lactante menor

**DOI:** 10.47487/apcyccv.v1i4.85

**Published:** 2020-12-31

**Authors:** Ángel Cueva-Parra, Fernando Taipe-Carbajal, Deli Guillén-Buleje, Silvia Alegre-Manrique

**Affiliations:** 1 Servicio de Electrofisiología - Instituto Nacional de Cardiología Ignacio Chávez. Ciudad de México, México. Servicio de Electrofisiología Instituto Nacional de Cardiología Ignacio Chávez Ciudad de México México; 2 Servicio de Cardiología - Instituto Nacional de Salud del Niño. Lima, Perú. Servicio de Cardiología Instituto Nacional de Salud del Niño Lima Perú; 3 Servicio de Pediatría - Instituto Nacional de Salud del Niño. Lima, Perú. Servicio de Pediatría Instituto Nacional de Salud del Niño Lima Perú

**Keywords:** Síndrome de Wolff-Parkinson-White, Lactante, Arritmias Cardíacas, Wolff-Parkinson-Wihte Syndrome, Infant, Arrhythmias, Cardiac

## Abstract

El síndrome de Wolff Parkinson White ocurre por la presencia de vías accesorias que comunican anormalmente las aurículas con los ventrículos, es una de las principales causas de taquicardia paroxística supraventricular en jóvenes y adolescentes, en quienes el manejo ideal es la ablación con catéter. Este síndrome también puede presentarse en pacientes de menor edad como neonatos y lactantes, en donde las opciones terapéuticas son distintas. Presentamos el caso de una paciente de 47 días de vida que ingresó a un hospital pediátrico público de Perú presentando vómitos, a su ingreso mostró una frecuencia cardiaca de 250 latidos por minuto; se logró documentar taquicardia de complejos QRS anchos; posteriormente, en el electrocardiograma en ritmo sinusal, se evidenció signos de preexcitación.

## Abstract (en)

Wolff Parkinson White Syndrome occurs due to the presence of accessory pathways that abnormally communicate the atria with the ventricles. It is one of the main causes of paroxysmal supraventricular tachycardia in young people and adolescents, in whom the ideal management is catheter ablation. This syndrome can also occur in younger patients such as neonates and infants, where the therapeutic options are different. We present the case of a 47-day-old patient who was admitted to a public pediatric hospital in Peru due to vomiting. Upon admission, she showed a heart rate of 250 beats per minute; wide QRS complex tachycardia was documented; later, on the electrocardiogram in sinus rhythm, signs of pre-excitation were evident.

El síndrome de Wolff - Parkinson - White (WPW) es la segunda causa más frecuente de taquicardia paroxística supraventricular (TPSV) ^1^^,^^2^. Para su diagnóstico es necesario un electrocardiograma (ECG) en ritmo sinusal donde se evidencia signos de preexcitación y, además, documentar la taquicardia ^2^^,^^3^. Las taquicardias generadas por una vía accesoria (VA) son conocidas como taquicardias por reentrada aurículo ventricular (TRAV) la cual puede ser ortodrómica, cuando la conducción anterógrada ocurre a través del nodo aurículo ventricular (AV) y la retrógrada a través de una VA; es antidrómica cuando la conducción a través de estas estructuras ocurre en sentido contrario ^2^^,^^3)^. El patrón de preexcitación tiene una prevalencia de 0,1 a 0,3% en la población general ^3^, en los lactantes y preescolares varía entre el 0,4 a 1 por 1000, encontrándose la mayoría en menores de 2 años ^4^. Presentamos el caso de una lactante menor con síndrome de WPW la cual se presentó como taquicardia de complejos QRS anchos.

## Presentación del caso

**Figura 1:**
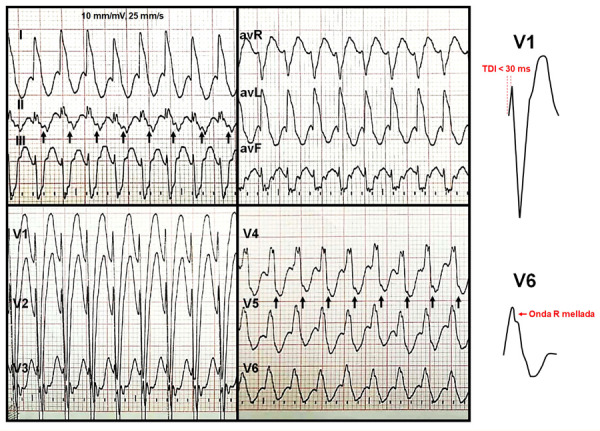
Electrocardiograma de doce derivadas que muestra taquicardia QRS ancho, RR regular con imagen típica de bloqueo de rama izquierda (morfología rS en V1 y R mellada en V6) y RP corto. Las flechas señalan las ondas p.

Paciente de sexo femenino de 47 días de vida, nacida por parto eutócico a término, sin antecedentes de importancia, que ingresó por emergencia presentando vómitos e irritabilidad, con frecuencia cardiaca de 250 latidos por minuto; se documentó en el ECG taquicardia QRS ancho, RR regular con una relación PR igual a 1 e intervalo RP corto **(**Figura 1**)**.

Este primer ECG tenía una morfología típica de bloqueo completo de rama izquierda (BCRI): en V1 se evidenciaban complejos rS con un tiempo de activación corto y en V6 presentaba complejos monofásicos positivos con onda R mellada. Esta morfología típica orienta al diagnóstico de una TPSV con fenómeno de aberrancia.

**Figura 2:**
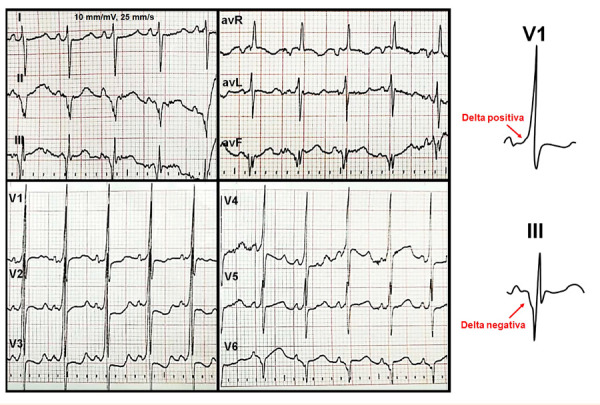
Electrocardiograma de doce derivadas que muestra ritmo sinusal con preexcitación por una vía accesoria posterior izquierda (onda delta negativa en III y positiva en V1).

Debido a la presencia de signos de bajo gasto la paciente fue cardiovertida eléctricamente con 5 J bajo sedación, pasando a ritmo sinusal en donde se apreció signos de preexcitación: intervalo PR corto con presencia de onda delta y alteraciones de repolarización **(**Figura 2**)**. El ecocardiograma mostró ausencia de cardiopatía estructural. Posteriormente, fue dada de alta con indicación de propafenona; en el control a los tres meses se mantenía asintomática en ritmo sinusal, pero persistía con presencia de onda delta.

## Discusión

La presentación clínica del síndrome de WPW depende de la edad y de la duración del cuadro, los lactantes cursan con diaforesis, palidez, astenia, vómitos, y tendencia al sueño o irritabilidad; si la taquicardia es persistente y dura más de 24 h podrían presentarse síntomas de insuficiencia cardiaca ^5^. El síndrome de WPW siempre obliga a descartar cardiopatía estructural puesto que la presencia de una VA puede asociarse con anomalías de las válvulas auriculoventriculares ^2^, en nuestro caso solo se encontró foramen oval permeable.

**Figura 3:**
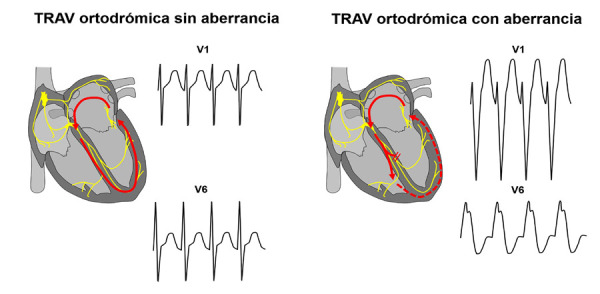
Diferencias electrocardiográficas en V1 y V6 durante la TRAV ortodrómica por una vía accesoria izquierda sin y con fenómeno de aberrancia de rama izquierda. Cuando ocurre la aberrancia el RR se incrementa discretamente

Si bien la mayoría de TRAV ortodrómicas son de QRS angosto, hay excepciones; en este caso la taquicardia tenía RP corto y QRS ancho, con imagen típica de BCRI en donde el tiempo de activación o deflexión intrínsecoide (TDI) en V1 era menor de 30 ms, lo cual excluye la posibilidad de taquicardia ventricular o una TRAV antidrómica **(Figura 1)**; por lo tanto, la taquicardia de nuestro paciente se trata de una TRAV ortodrómica con aberrancia, la cual es un fenómeno que consiste en el bloqueo funcional de una de las ramas del haz de His secundario a una frecuencia cardiaca muy elevada. Cuando ocurre este fenómeno es muy probable que la VA sea ipsilateral a la rama bloqueada ^3^; en este caso, la TRAV presentaba aberrancia de rama izquierda y la ubicación probable de la VA determinada por ECG era izquierda **(**Figura 3**)**.

En 1996 Iturralde *et al.* elaboraron un algoritmo para ubicar la vía accesoria en pacientes con síndrome de WPW, este se basaba en la polaridad del QRS durante el ritmo sinusal y es mucho más útil aún en pacientes con preexcitación máxima, es decir, cuando la conducción anterógrada ocurre de manera preferencial por la VA ^6^. Los hallazgos electrocardiográficos de la preexcitación máxima son: presencia de onda delta clara, QRS ensanchado y alteraciones de la repolarización. La paciente en mención tenía en ritmo sinusal una onda delta clara y QRS de 100 ms el cual sobrepasaba el percentil 98 para la edad, por lo tanto, se podría catalogar como ancho para su edad. En cuanto a la polaridad de la onda delta, esta era negativa en DIII y positiva en V1, lo cual sugiere que la ubicación de la VA es posterior izquierda no descartándose una ubicación dentro de seno coronario por presentar onda delta negativa en DII.

En cuanto al manejo agudo de la TPSV la primera opción es el uso de adenosina, la cual, al bloquear el nodo AV y ser este parte del circuito, interrumpe la arritmia. Si fallara o hay inestabilidad hemodinámica se debe realizar cardioversión eléctrica sincronizada ^2^^,^^3^. En cuanto al manejo crónico, la ablación con catéter no es de primera elección en pacientes con menos de quince kilogramos, salvo que la arritmia sea incesante. Usualmente a niños pequeños se les debe ofrecer manejo médico, en primera instancia, con antiarrítmicos (de preferencia bloqueadores de canales de sodio asociados o no a betabloqueadores) y seguimiento para valorar la necesidad de ablación en el futuro cuando tengan mayor edad y peso. Finalmente, cabe destacar que hay casos en los cuales el patrón de preexcitación puede desaparecer a medida que el paciente crece ^1^^,^^4^. 
